# Long-Term Spatiotemporal Variations in Soil Moisture in North East China Based on 1-km Resolution Downscaled Passive Microwave Soil Moisture Products

**DOI:** 10.3390/s19163527

**Published:** 2019-08-12

**Authors:** Xiangjin Meng, Kebiao Mao, Fei Meng, Xinyi Shen, Tongren Xu, Mengmeng Cao

**Affiliations:** 1 School of Surveying and Geo-Informatics, Shandong Jianzhu University, Jinan 250100, China; 2 School of Geography, South China Normal University, Guangzhou 510631, China; 3 Institute of Agricultural Resources and Regional Planning, Chinese Academy of Agricultural Sciences, Beijing 100081, China; 4 State Key Laboratory of Remote Sensing Science, Institute of Remote Sensing and Digital Earth Research, Chinese Academy of Science and Beijing Normal University, Beijing 100101, China; 5 Civil and Environmental Engineering, University of Connecticut, Storrs, CT 06269, USA

**Keywords:** soil moisture, passive microwave, downscaled, anomaly analysis

## Abstract

It is very important to analyze and monitor agricultural drought to obtain high temporal-spatial resolution soil moisture products. To overcome the deficiencies of passive microwave soil moisture products with low resolution, we construct a spatial fusion downscaling model (SFDM) using Moderate Resolution Imaging Spectroradiometer (MODIS) data. To eliminate the inconsistencies in soil depth and time among different microwave soil moisture products (Advanced Microwave Scanning Radiometer on the Earth Observing System (AMSR-E) and its successor (AMSR2) and the Soil Moisture Ocean Salinity (SMOS)), a time series reconstruction of the difference decomposition (TSRDD) method is developed to create long-term multisensor soil moisture datasets. Overall, the downscaled soil moisture (SM) products were consistent with the in situ measurements (R > 0.78) and exhibited a low root mean square error (RMSE < 0.10 m^3^/m^3^), which indicates good accuracy throughout the time series. The downscaled SM data at a 1-km spatial resolution were used to analyze the spatiotemporal patterns and monitor abnormal conditions in the soil water content across North East China (NEC) between 2002 and 2018. The results showed that droughts frequently appeared in western North East China and southwest of the Greater Khingan Range, while drought centers appeared in central North East China. Waterlogging commonly appeared in low-terrain areas, such as the Songnen Plain. Seasonal precipitation and temperature exhibited distinct interdecadal characteristics that were closely related to the occurrence of extreme climatic events. Abnormal SM levels were often accompanied by large meteorological and natural disasters (e.g., the droughts of 2008, 2015, and 2018 and the flooding events of 2003 and 2013). The spatial distribution of drought in this region during the growing season shows that the drought-affected area is larger in the west than in the east and that the semiarid boundary extends eastward and southward.

## 1. Introduction

Soil moisture (SM) is the primary indicator of climate change and has been defined as an essential climate variable by the Global Climate Observing Systems [1]. SM has been widely used to elucidate the water and energy cycles [2], predict meteorological conditions [3], monitor drought or flood disasters [4], and provide insights for water resource and agricultural irrigation management programs [5]. Although temporal and spatial information on SM can be obtained through multiple approaches, such as in situ measurements and remote sensing (RS) methods. However, the accuracy of in situ measurements of SM is limited by the number of field stations around the world. Measuring SM at a single location does not necessarily represent the conditions of the entire region, only RS can provide real-time dynamic observations at regional to global scales [6,7]. Over the past several decades, RS has become a practical method for deriving SM information, especially in remote countryside areas with limited ground measurements [8]. Several approaches for retrieving SM based on RS data exist, such as those based on visible/infrared (IR) and microwave observations [9].

Temperature/vegetation indexes for indirect SM detection based on visible/IR observations have been developed. The vegetation condition index (VCI) developed by Kogan (1990) has been used to describe drought conditions by comparing the current and vegetation conditions with those in the same period in previous years [10]. The anomaly vegetation index (AVI) is more accurate than the VCI because the AVI decreases the time lag between vegetation and precipitation measurements [11]. The temperature condition index (TCI), which was proposed by Kogan (1995), combines the VCI and the TCI to estimate soil drought from brightness temperature values [12]. Additionally, the vegetation supply water index (VSWI) is based on the relationship between the normalized difference vegetation index (NDVI) and the land surface temperature (LST) [13]. Sandholt et al. (2002) utilized the NDVI-LST triangular space and developed the temperature-vegetation dryness index (TVDI) and found that the TVDI was significantly negatively correlated with SM [14]. Although optical sensors can reflect the dry and wet conditions of the soil on local and basin scales, they are affected by factors such as the atmosphere, clouds, vegetation coverage, and terrain fluctuations [15]. Microwave RS data can estimate SM information through the stark contrast in microwave bands between the dielectric constant of soil and water [16]. Microwave RS data can estimate SM information through the stark contrast in microwave bands between the dielectric constant of soil and water. Microwave RS not only is less affected by cloud coverage but also can be used to obtain high-precision SM data over bare soil and low vegetation coverage areas [17,18,19,20]. Low-frequency microwaves can work in the dark, providing a basis for studying the diurnal changes in SM and the resulting impacts. Since the late 1970s, various microwave radiometers [e.g., the scanning multichannel microwave radiometer (SMMR), the special sensor microwave/imager (SSM/I), tropical rainfall measuring mission (TRMM) microwave imager (TMI), Sentinel-1 and the Advanced Microwave Scanning Radiometer on the Earth Observing System (AMSR-E) and its successor (AMSR2)], which operate in the C-band (or at a higher frequency), have been utilized to retrieve SM data [21,22,23]. The SMOS and soil moisture active passive (SMAP) sensors operate in the L-band to estimate SM [16,24,25]. However, microwave SM products with coarse spatial resolution (especially passive microwave sensors with spatial resolutions typically less than approximately 25 km) may not have been suitable for regional-scale applications (agriculture, etc.), which typically require SM products with fine spatial resolution (1~10 km) [26,27]. To improve the spatial resolution of SM data, several disaggregation approaches have been developed to downscale the coarse-spatial-resolution SM data obtained from passive microwave satellites [28,29]. One downscaling method uses active microwave backscatter observations to downscale passive microwave SM products [30,31]. The principle behind this method is based on the relationships among the brightness temperature, backscattering coefficient and surface SM and the vegetation status to establish a forward model, thereby determining the difference between simulated data and satellite observations and minimizing the cost function through an iterative process [32,33]. In addition, several studies have used the relationship between high-resolution visible/IR data and coarse-resolution passive microwave SM data for downscaling [34,35,36,37]. The well-known universal triangle formed by the feature space of LST over areas with heterogeneous NDVI has been confirmed as a feasible approach for downscaling SM data [38]. Ray et al. (2010) adopted a similar method to downscale 25-km AMSR-E SM products to a 1 km spatial resolution in Cleveland Corral, California [39]. Peng et al. (2016) also downscaled low-resolution SM estimates using a vegetation temperature condition index (VTCI) [40].

The abovementioned studies are mostly based on a single microwave sensor that is limited to a specific area within a restricted time, as determined by the life of the satellite sensor. Therefore, it is very difficult to acquire accurate long-term SM data over a relatively large area at fine spatial resolution. Here, to obtain long-term and high-resolution multisensor SM datasets, we construct a downscaling model that directly uses MODIS LST/NDVI data as the input. A time series reconstruction of the difference decomposition (TSRDD) method is developed to capture long-term multisensor SM datasets to ensure the consistency of long-term sequence data at different times. The downscaled SM results were evaluated by ground measurements. Furthermore, we analyzed the monthly daytime and nighttime 1-km SM anomalies from 2002 to 2018 in North East China based on the downscaled products.

## 2. Materials and Methods

### 2.1. Study Area

**Figure 1:**
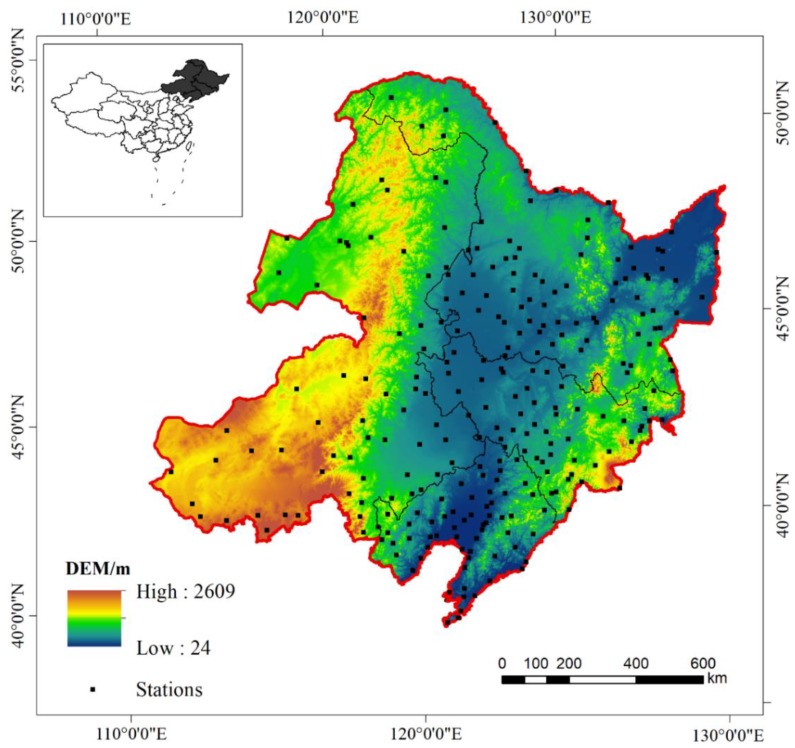
The location and topography of the study area and 262 ground meteorological sites in the study area where data were collected.

North East China (38°42′N~53°34′N, 117°16′E~135°21′E) is located in the high-latitude region of eastern Eurasia, spanning four provinces, including Heilongjiang, Jilin, Liaoning and the northeastern part of Inner Mongolia (Figure 1), and covers an area of 16.13 × 10^5^ km^2^. North East China belongs to the moderate-temperature and cold-temperate monsoon climate zones, with a mean annual temperature ranging from approximately −4 to 11 °C. The mean annual rainfall decreases from the southeast (1000 mm) to the northwest (300 mm), spanning from the wet zone to the semihumid and semiarid zones [41]. All of these annual averages were derived from weather stations in the study area in 2016. The soil types in North East China are dominated by high-fertility black soil (the average organic content is between approximately 3% and 10%). The vegetation types include cold-temperate coniferous forests distributed in the northern part of the Greater Xing’an Mountains, coniferous and broad-leaved mixed forests in the eastern mountains, and temperate forests and grasslands in the central plains. The main land cover types in the region are forestland, dryland, paddy fields, and grassland, accounting for 41.50%, 27.78%, 3.62%, and 18.53% of the total area, respectively [42]. The study area is centered on the Songnen Plain, and a horseshoe-shaped landform pattern is formed in the east, north, and west. The northern part of the Greater Xing’an Mountains in the northwest includes a series of mountain ranges: Xiaoxing’anling in the north, Daheishan in the middle, Hadaling in Jilin Province, and Zhangguangcailing, Laoyeling, Wandashan, and Changbai in the south. Typical dry-wet differentiation, a unique vegetation distribution, and relatively complete natural geographic regions, combined with seasonal interdecadal features of seasonal precipitation and temperature, are closely related to the occurrence of extreme weather events (such as droughts and floods); thus, North East China has become a hot spot for global climate change and ecological geography research [43].

### 2.2. Datasets

#### 2.2.1. LST and NDVI

We used the LST and NDVI RS products retrieved from MODIS, which is onboard the Aqua satellite; these products have been widely used to monitor surface, oceanic, and atmospheric conditions [44,45]. Aqua passes over North East China at approximately 01:30 a.m. (descending) and 01:30 p.m. (ascending). In this study, the MODIS products were obtained from National Aeronautics and Space Administration’s (NASA’s) Earth Observing System Data and Information System. The 1-km, 8-day composite LST (day/night) product MYD11A2 and the 1-km 16-day composite NDVI product MYD13A3 were used as inputs in the TVDI model.

#### 2.2.2. Soil Moisture (SM)

The soil moisture data (humidity unit: m^3^/m^3^) were mainly derived from the Level-3 products of AMSR-E, SMOS, and AMSR2, with resolutions of 25, 25, and 10 km, respectively. The AMSR-E sensor is mounted on the Aqua satellite, and its orbital parameters are consistent with those of MODIS. Hence, the AMSR-E Level 3 products were included in the analysis for both day (01:30 p.m.) and night (01:30 a.m.) overpasses. The time span was from July 2002 to September 2011, and the data were provided by the Japan Aerospace Exploration Agency (JAXA, http://global.jaxa.jp/projects/sat/gcom_w/index.html). The AMSR-E SM products were retrieved using the vertically and horizontally polarized brightness temperature (Tb) from the C-band (7.3 GHz) and the X-band (10.7 GHz). The AMSR2 instrument is onboard the Global Change Observation Mission–Water 1 (GCOM-W1) satellite, which was launched by JAXA in May 2012 and measures C-band and X-band emissions with overpass times of 01:30 a.m. and 01:30 p.m. [46]. As a successor to AMSR-E, AMSR2 provides near-real-time passive microwave observations [47]. The AMSR2 Level 3 SM data were provided by JAXA’s GCOM website (http://suzaku.eorc.jaxa.jp/).

The European Space Agency’s (ESA’s) SMOS mission, which was launched in 2009 as part of the Living Planet Programme, was used to study the L-band (1.4 GHz) emissions. The transit times of the ascending and descending orbits are at approximately 06:00 a.m. and 06:00 p.m., respectively, in local time (~3 days), and the study period spanned from October 2011 to June 2012. While the original resolution of the SMOS SM data is 35 to 55 km, the Level 3 products were provided on the Equal Area Scalable Earth (EASE) grid, with a 25-km sampling interval [48]. The data were acquired from ESA’s official website (https://earth.esa.int/). The monthly 25-km resolution SMOS Level-3 product integrates the averages of daytime and nighttime data but does not include individual values for day and night. To make the products consistent, the AMSR-E, AMSR2, and SMOS products were corrected with the time series reconstruction method.

#### 2.2.3. Meteorological Data

In situ SM records were collected from 24-h field stations in North East China from the Soil Moisture on the Growth and Development of Crops in China dataset (http://data.cma.cn/site/index.html, last access: 28 February 2019) provided by the China Meteorological Science Data Service Network (CMSDSN). The in situ SM data were compared with the results of the downscaled SM calculation, and weather anomalies and nonrepresentative data were excluded (Figure 1). Ground SM data were measured at approximately 1:30 p.m. and 1:30 a.m. (local solar time), corresponding to the satellite overpass time. These data were used and compared with the corresponding downscaled daytime and nighttime SM data. The ground-based SM measurements were matched with the grid points of the downscaled SM data using the nearest neighbor approach. In this study, the verification work was carried out on a monthly scale, so the monthly ground station aggregate data were averaged from the daily meteorological site data that were selected.

## 3. Methods

**Figure 2:**
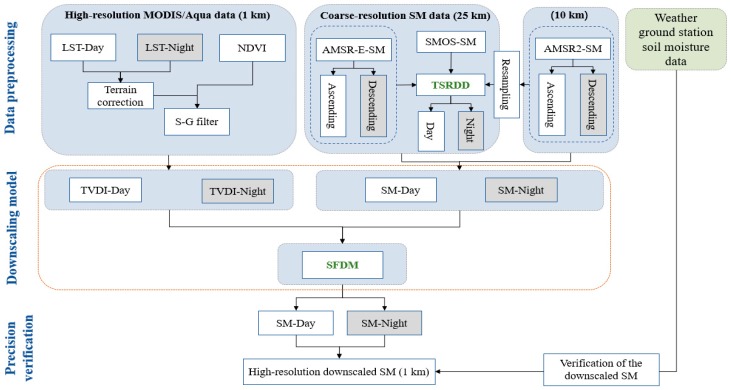
Flow chart of the downscaling process.

A summary of the downscaling method is shown in Figure 2, and additional details are described in the following sections.

### 3.1. Calculation of the TVDI

Theoretically, the temperature will decrease by 6 °C for every 1-km increase in elevation [49], and the elevation difference between the highest and lowest areas in North East China is greater than 2 km. Therefore, we need to correct the MODIS surface temperature products to reduce the impact of elevation on the surface temperature inversion before calculating the TVDI:

*T_m_* = *T_s_* + *a* * *h* (1)

where Tm is the LST after topographic correction, Ts is the surface temperature before topographic correction, and h is the elevation value at the LST pixel. The temperature variables are in °C. The variable a is the average influence coefficient of elevation on the LST inversion process (the common value is 6 °C/km).

In practice, due to weather conditions, there are some missing deviations in some time series data; to compensate for data errors caused by weather conditions, a Savitzky-Golay (S-G) filter timing reconstruction method is applied in this study to improve the quality of the data. The formula for processing the data via S-G filtering is as follows:

(2) $$Y_{j}^{*}=\sum_{i=-m}^{i=m}\frac{C_{i}Y_{j+1}}{N}$$

where Y_j_^*^ is the filtered reconstructed data, *Y_j+_*_1_ is the original time series data, m is the size of the moving filter window, *C_i_* is the fitting coefficient of the S-G filter polynomial, that is, the *i-th* weight index starting from the filter header of each value, and *N* is the length of the filter processing data.

The TVDI values were calculated as follows [47]:

*TVDI* = *T_m_* − *T_smin_*/(*T_smax_* − *T_smin_*) (3)

where *TVDI* is the temperature-vegetation dryness index, Tm represents the LST after topographic correction, *T_smax_* is the highest surface temperature on the dry edge corresponding to the NDVI, and *T_smin_* is the lowest surface temperature on the wet edge corresponding to the NDVI. We can calculate *T_smax_* and *T_smin_* as follows:

*T_smax_* = *a_1_* + *b_1_* * *NDVI* (4)

*T_smin_* = *a_2_* + *b_2_* * *NDVI* (5)

where *a_1_* and *b_1_* represent the slope and intercept on the “dry edge”, respectively, and *a_2_* and *b_2_* represent the slope and intercept on the “wet edge”, respectively. NDVI is the normalized difference vegetation index in the study time series (from 2002 to 2018).

### 3.2. Time Series Reconstruction of Difference Decomposition (TSRDD) Method

The SMOS Level 3 products used in this study are monthly averages of the daytime and nighttime values, and the SMOS and AMSR series satellites have different descending and ascending times. To ensure data consistency and integrity, the time series reconstruction with difference decomposition (TSRDD) method is developed according to the time series principles to capture long-term daytime and nighttime SM series datasets at different times. The TSRDD method includes a consistency correction and generates the daytime and nighttime data from the SMOS series data, thereby obtaining a long-term, high-precision SM dataset that ensures consistency among the multisensor SM data at different times.

Ẑ(x, j) _day_ = Ẑ(x, j)_average_ + ΔF_j_ (6)

*Ẑ*(*x*, *j*) *_night_* = *Ẑ*(*x*, *j*)*_average_* − *ΔF_j_* (7)

(8) $$\Delta F_{j}=\frac{\sum_{i=1}^{\frac{w}{2}}\left(F_{day\;i}-F_{night\;i}\right)+\sum_{i=1+\frac{w}{2}}^{w}\left(F_{day\;i}-F_{night\;i}\right)}{w-1}$$

where *Ẑ(x,j)*_day_ is the reconstructed daytime SM in month j of year x and *Ẑ(x,j)*_night_ is the reconstructed nighttime SM in month *j* of year *x*. $\Delta F_{j}$ represents the difference between daytime and nighttime (day minus night) in month *j*. *Ẑ(x, j)*_average_ is the average value in month *j* of year *x*, and w is the selected year window size.

### 3.3. Spatial Fusion Downscaling Model (SFDM) of SM

The TVDI is significantly negatively correlated with SM, as shown in previous studies [14]. According to this correlation, the high-resolution TVDI is used to perform the pixel-by-pixel weighting of low-resolution SM data. Then, the weight is used to divide the low spatial resolution SM product into high-spatial-resolution SM products, and the downscaling relationship is as follows:

(9) $$SM_{i}=SM_{k}\times \frac{1-TVDI_{i}}{1-TVDI_{average}}$$

where *SM_i_* is the SM value for a pixel after downscaling and *SM_k_* represents the low-resolution SM data input. *TVDI_i_* is the TVDI value for a pixel that corresponds to the downscaled SM. *TVDI_average_* is the average of all the TVDI values corresponding to the *SM_k_* pixels.

### 3.4. The Calculation of the Trend of the Variation in SM

To analyze the change (increase or decrease) in the spatial pattern and the amplitude of the SM from 2002 to 2018, the variation rates and correlation coefficient were calculated based on the SM values of the pixels over the study area as follows:

(10) $$Slope=\frac{n\sum_{i=1}^{n}\left(i\times p_{i}\right)\times \left(\sum_{i=1}^{n}i\right)\times \left(\sum_{i=1}^{n}p_{i}\right)}{\left(n\times \sum_{i}^{n}i^{2}\right)-{\left(\sum_{i=1}^{n}i\right)}^{2}}$$

(11) $$R=\frac{n\sum_{i=1}^{n}\left(i\times p_{i}\right)-\left(\sum_{i=1}^{n}i\right)\times \left(\sum_{i=1}^{n}p_{i}\right)}{\sqrt{\left(n\sum_{i}^{n}i^{2}\right)-{\left(\sum_{i=1}^{n}i\right)}^{2}}\times \sqrt{\left(n\sum_{i}^{n}p_{i}{}^{2}\right)-{\left(\sum_{i=1}^{n}p_{i}\right)}^{2}}}$$

where *Slope* is the trend in the SM during the study time series, *i* represents the number of years, *n* represents the number of time series, and *p_i_* represents the SM value of the *i-th* year. If *Slope* is positive, then the SM has increased from the previous time point. If *Slope* is negative, then the SM has decreased from the previous time point. If *Slope* = 0, then there is no change from the previous time point. *R* is the correlation coefficient between the change in the SM and the time in the time series. The larger the absolute value of *R* is, the stronger the correlation.

### 3.5. Soil Moisture Anomaly (SMA) Index

The soil moisture anomaly (SMA) index is used to describe dramatic changes in the SM in the study region (too low or too high), and the SMA index can be derived from the following formula [27]:

*SMA_i(x, y)_* = *(SM_i(x, y)_* − *SM_min(x, y)_)/(SM_max(x, y)_* − *SM_min(x, y)_) × 100%* (12)

where *SMA_i__(x, y)_* is the SMA index of pixel (*x, y*) at time *i*, *SM_i__(x, y)_* is the SM value of pixel (*x, y*) at time *i*, *SM_max__(x, y)_* is the maximum SM value of pixel (*x, y*) during the study period, *SM_min__(x, y)_* is the maximum SM value of pixel (*x, y*) during the study period, and *SM_average__(x, y)_* is the average SM value of pixel (*x, y*) during the study period.

### 3.6. Drought and Flood Degree

To characterize the spatiotemporal distribution of drought and waterlogging over the 198 months from 2002 to 2018 (excluding January to June 2002), the SMA index was divided into seven grades based on the literature concerning drought and waterlogging classification standards: slight waterlogging (SLW), moderate waterlogging (MOW), normal (no disasters), severe waterlogging (SEW), slight drought (SLD), moderate drought (MOD), and severe drought (SED), as shown in Table 1.

## 4. Results

### 4.1. Verification

#### 4.1.1. TSRDD Verification

To ensure the consistency of the data between different sensors and obtain the SMOS daytime and nighttime data, the most suitable timing window is selected. The SM value is normally distributed in the study area, which satisfies the basic conditions for the correlation analysis between the simulated, corrected, and original values. The relationship between the corrected variables and explanatory variables (original value) varies significantly for different window sizes (*w*). The regression coefficients (root mean squared error (RMSE), mean absolute error (MAE), and *R^2^*) of the TSRDD are presented in Table 2.

For daytime, the indexes are best matched when *w* is 5: the RMSE (m^3^/m^3^), MAE (m^3^/m^3^), and *R^2^* are 2.11, 1.46, and 0.96, respectively. For nighttime, the indexes are best matched when *w* is 3: the RMSE (m^3^/m^3^), MAE (m^3^/m^3^), and *R^2^* are 2.07, 1.56, and 0.95, respectively. Therefore, these two windows are used to decompose the SM data from the SMOS daytime and nighttime data.

#### 4.1.2. Downscaled SM Verification

**Figure 3:**
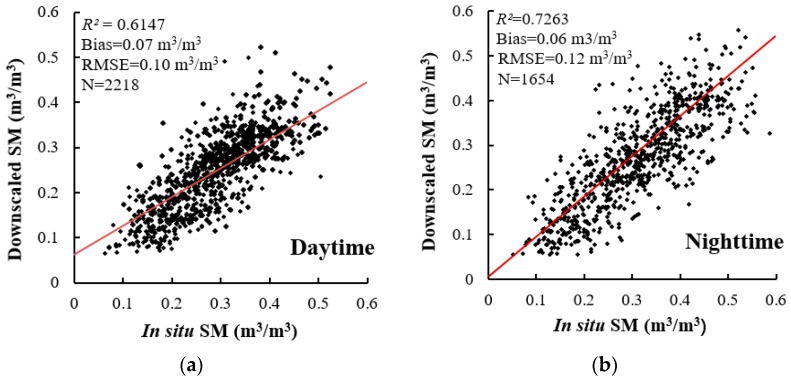
Downscaled soil moisture (SM) (1-km scale) based on the spatial fusion downscaling model (SFDM) versus the in situ SM: (**a**) daytime; (**b**) nighttime. The solid line is the trend line.

To quantitatively evaluate the performance of daytime and nighttime downscaling, considering the effects of weather factors, we compared the downscaled passive microwave SM values (at the 1-km scale) with meteorological in situ SM observations scattered throughout the North East China area for daytime and nighttime, and the *R^2^*, RMSE, and bias values were used for the comparison. The results showed that the downscaled and in situ observed SM were strongly correlated, with mean *R^2^* values of 0.61 and 0.73 for daytime and nighttime, respectively (Figure 3). The mean RMSE and bias values were 0.10 m^3^/m^3^ and 0.07 m^3^/m^3^ for daytime and 0.12 m^3^/m^3^ and 0.06 m^3^/m^3^ for nighttime, respectively. The results indicated a significantly high correlation between the downscaled passive microwave and in situ measured SM data during the daytime (Figure 3a) and nighttime (Figure 3b).

**Figure 4:**
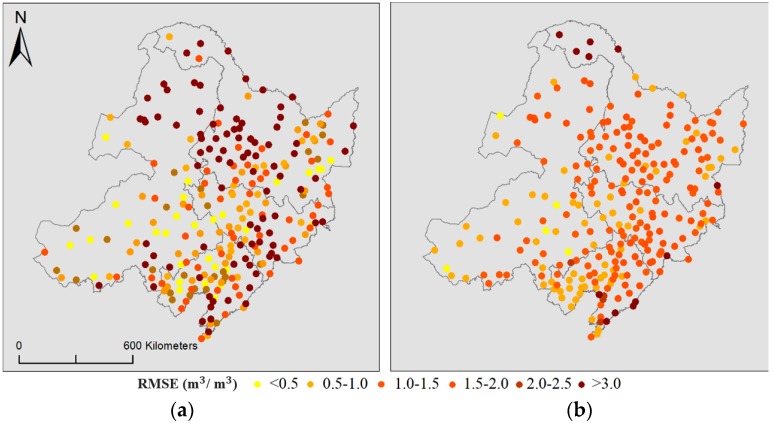
Spatial variations in the RMSE for each meteorological station in the study area in the study time series for (**a**) daytime and (**b**) nighttime.

Figure 3 and Figure 4 display the accuracy analysis and show significant spatiotemporal differences between day and night. First, the *R^2^* values of the nighttime observations were slightly higher than those of the daytime observations. Second, the points were more aggregated at night than during the day. One possible reason for the increased nighttime accuracy may be the difference in the time lag between the downscaled SM and in situ observations. Similar results were reported for LST and measured temperature [50]. The accuracy analysis also presented a certain spatial differentiation: The RMSE was high along rivers and coasts, as well as in some mountainous areas (>0.3), in the daytime, and the RMSE was low (<0.1) in the plain regions. Moreover, the spatial differentiation characteristics at night were less obvious than those during the day (except for several stations along the boundary).

### 4.2. Spatiotemporal Variation in the SM

**Figure 5:**
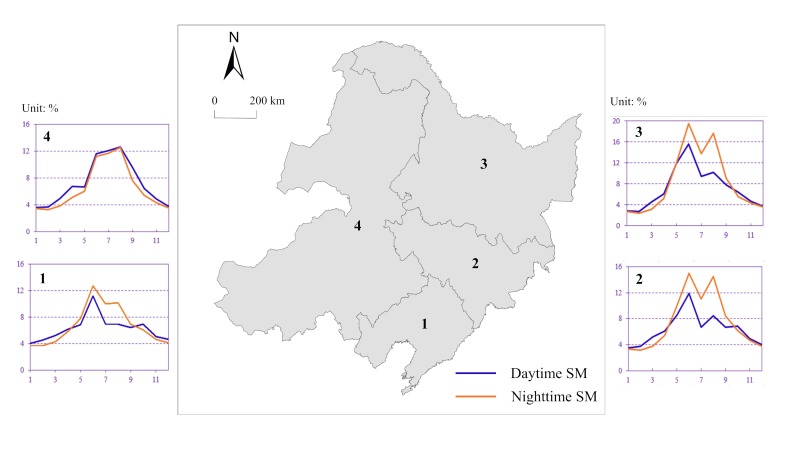
Mean monthly daytime and nighttime downscaled SM from January to December in various parts of North East China (NEC). 1: Liaoning, 2: Jilin, 3: Heilongjiang, 4: northeastern Inner Mongolia.

The downscaled SM showed significant spatiotemporal variations spanning different seasons and regions. We aggregated the downscaled SM by month for four regions in North East China (Figure 5). The mean monthly daytime SM in the four regions ranged from approximately 0.03 m^3^/m^3^ (February in Heilongjiang) to 0.16 m^3^/m^3^ (June in Heilongjiang). Similarly, the mean monthly nighttime SM ranged from 0.03 m^3^/m^3^ (February in Heilongjiang) to 0.20 m^3^/m^3^ (June in Heilongjiang). The downscaled SM also showed significant temporal patterns. First, there were apparent seasonal variations in the SM values, with high values in summer and low values in winter (Figure 5). There was an east-west distribution pattern, and in the eastern region, there was a valley in July (summer). One possible reason for the “bimodal” distribution in the eastern region could be that the distance between land and sea and the sinking airflow from the Greater Xing’an Mountains reduced precipitation in the coastal areas in July.

**Figure 6:**
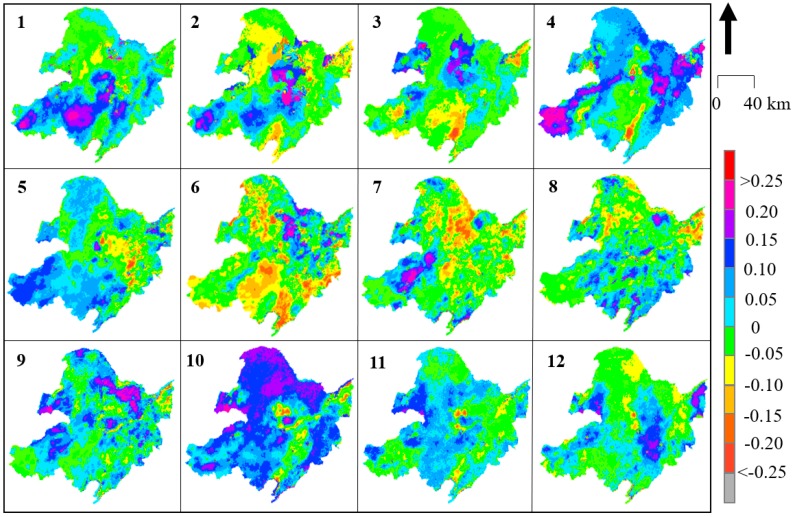
Trends in *Slope* for the mean monthly downscaled daytime SM from 2002 to 2018 in NEC.

**Figure 7:**
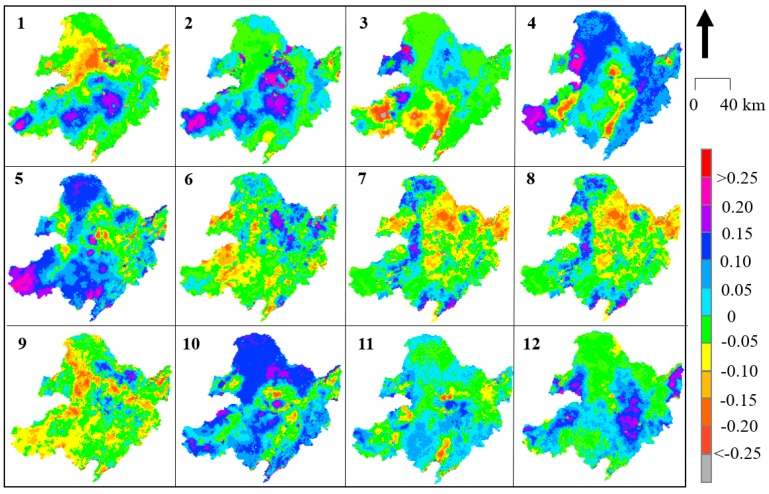
Trends in *Slope* for the mean monthly downscaled nighttime SM from 2002 to 2018 in NEC.

**Figure 8:**
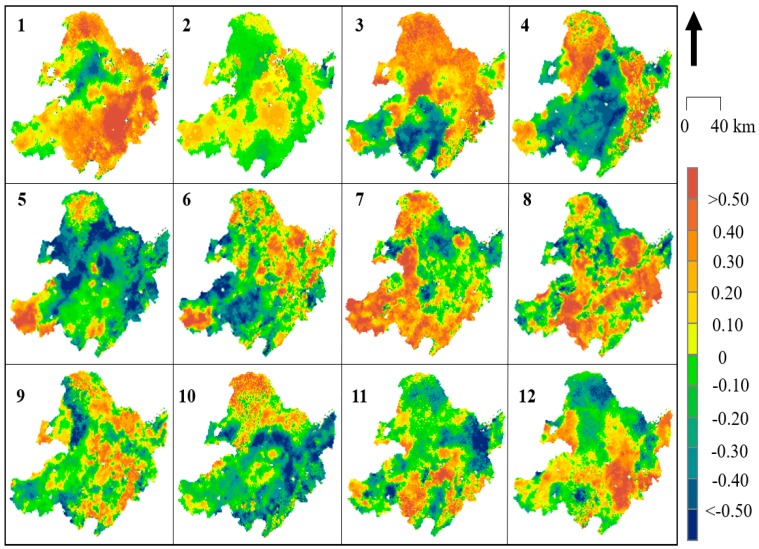
Trends in *R* for the mean monthly downscaled daytime SM from 2002 to 2018 in NEC.

**Figure 9:**
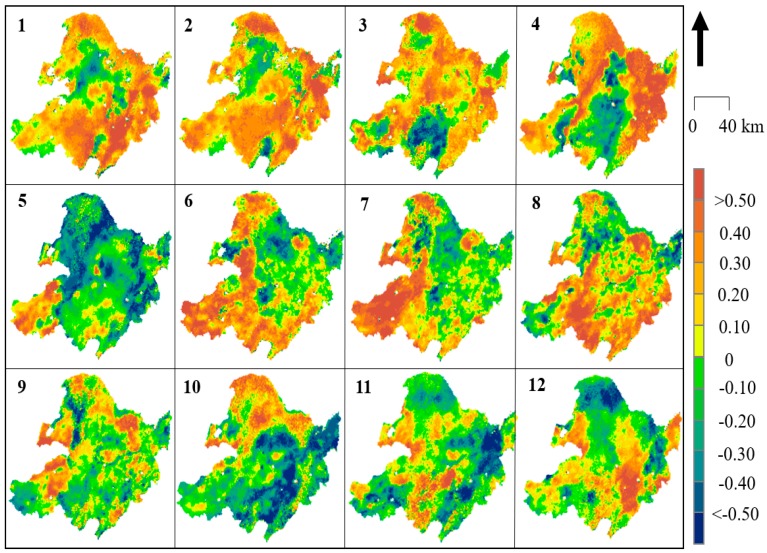
Trends in *R* for the mean monthly downscaled nighttime SM from 2002 to 2018 in NEC.

To obtain the spatiotemporal rate of change in the downscaled SM in North East China in detail, formulas (9) and (10) were utilized to calculate the monthly changes using every pixel from 2002 to 2018 (Figure 6 and Figure 7 represent the *Slope* values for daytime and nighttime, respectively; Figure 8 and Figure 9 represent the *R* values for daytime and nighttime, respectively). As shown in Figure 6, the SM in the North East China region significantly increased over the past 17 years, with obvious monthly differences. The SM distribution in North East China was more stable at night than during the day. The most significant changes in SM during the day occurred in spring (April) and autumn (October), and the two trends were consistent. This “correspondence phenomenon” is thought to be related to the interaction between vegetation and climate (this trend is present during both the day and night). The SM variation in North East China exhibited obvious seasonality. In spring (March, April, and May), the southwestern region (particularly in the Inner Mongolia area) suffered from the highest increasing trend, with an increasing *Slope* of more than 0.3 (*R* > 0.20) that extended over 35.7% of the Xiaoxing’anling area. The increase was also greater than 0.3 (*R* > 0.25) in the Songnen Plain and the Sanjiang Plain in April (in the daytime). The variation in summer (June, July and August) was relatively uniform, and the *Slope* value of the change was within 0.2 (in the daytime and nighttime). Almost everywhere across North East China, except for a few mountain areas, the *Slope* value decreased, including in the southern Greater Xing’an Mountains (less than −0.2), while the *Slope* value south of Xiaoxing’anling increased (greater than 0.2), and the *Slope* was slightly higher in summer than in other seasons. There were dramatic changes in the spatial patterns of the SM values during autumn (September, October, and November). The SM values increased in the northwest (*Slope* > 0.20, *R* > 0.20) and decreased in the southeast (*Slope* < −0.20, *R* < −0.10). In winter (November, December, and January), the southern region centered at the Horqin Sandy Land exhibited an increase in *Slope* (>0.4), and the opposite was observed in summer (May: *Slope* < −0.3).

Overall, the consistencies of the daytime and nighttime changes in autumn and winter were better than those in spring and summer, and the consistency in the western region (northeastern Inner Mongolia) was better than that in the eastern region (Liaoning, Jilin, Heilongjiang). One possible reason for this phenomenon is that the vegetation density is high and complex in the east, the river network is dense, and the soil evapotranspiration changes greatly between day and night, so there will be large differences between daytime and nighttime.

### 4.3. Spatiotemporal Variations in SMA

**Figure 10:**
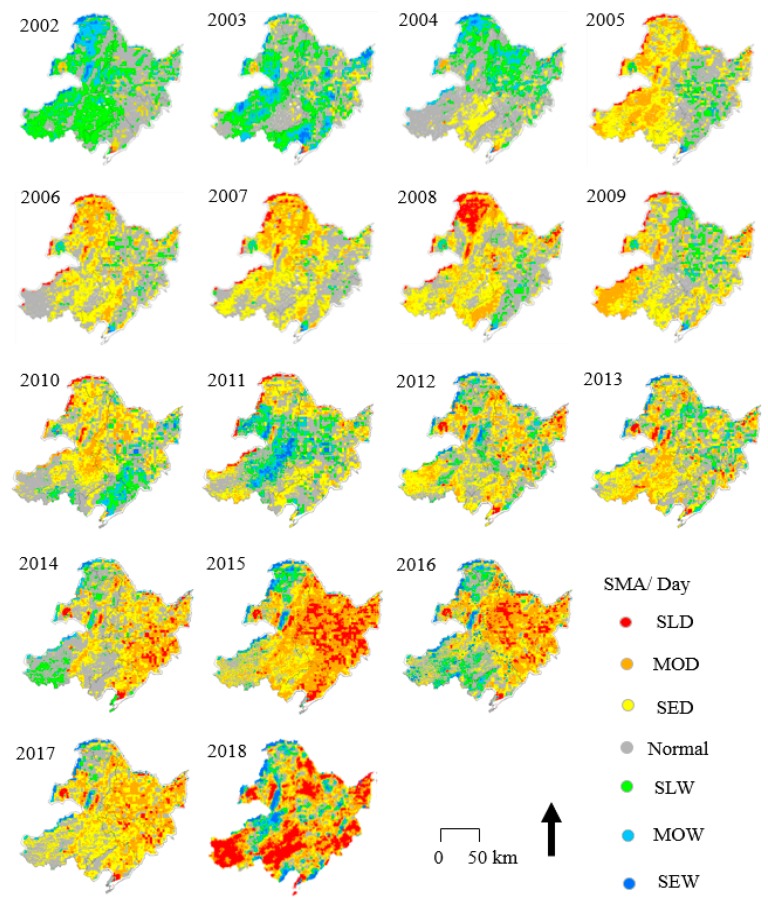
Spatial distribution of daytime soil moisture anomaly (SMA) in NEC from 2002 to 2018.

**Figure 11:**
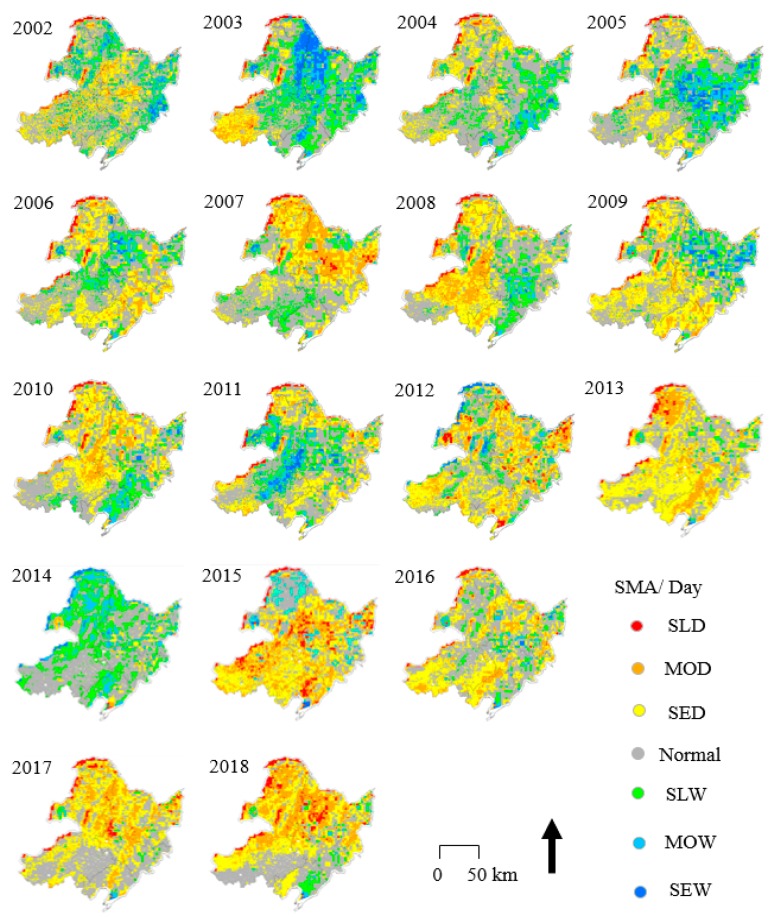
Spatial distribution of the nighttime SMA in NEC from 2002 to 2018.

Although the monthly variations in *Slope* and the correlation coefficient R show the spatial trends of SM (Figure 6, Figure 7, Figure 8 and Figure 9), some annual abnormalities need to be further analyzed. As shown in Figure 10 and Figure 11, the SMAs are mapped to investigate the spatial distribution of soil water anomalies during the daytime and nighttime over 17 years. The large-scale severe drought phase mainly occurred in three periods, 2008, 2015, and 2018. In 2008, a large-scale rain and snow disaster occurred in southern China, and the Wenchuan earthquake caused intense crustal movement. According to the SMA analysis, SLD conditions were mainly concentrated in the northern part of Inner Mongolia and the Hulunbeier Plateau in the west of Heilongjiang (daytime), and the area with SED conditions accounted for more than 80% of the total study area (average of daytime and nighttime). In this survey, the area with SED conditions accounted for 75% in 2015, that is, more than 3/4 of the total drought and drought risk area. In 2015, a large-scale drought occurred in the northeastern region. The drought in 2018 was consistent with the agricultural drought in the spring and summer (daytime and nighttime means). The large-scale and severe waterlogging phases mainly occurred in two periods, 2003 and 2014. The waterlogging situation in 2003 did not exhibit a wide spatial distribution, but it still caused abnormal changes within Bohai Bay (the affected area exceeded 4.82 × 104 km^2^). The SMA value of some regions increased in 2014, such as the Changbai Mountain area, but the flood trends became more obvious. Combined with a large amount of snowfall in the winter of 2013, there were some impacts on the accuracy of SM extraction in this area. Additionally, the daytime SMA exhibited increased variation, especially in the mountain areas, and this variation was greater than that in the nighttime SMA areas. The radiation is greater and active vegetation evapotranspiration and soil water evaporation are higher during the day than at night; however, these values also exhibit greater changes during the day than at night.

Snow (ice) coverage and low temperatures are mutually influential processes in winter. Snow accumulation leads to an increase in surface reflectivity, which reduces the radiant energy absorbed by the surface and decreases the temperature. The decreased temperature will reduce the speed of snow melting. Therefore, the large amount of snowfall in winter in recent years can be considered one of the main causes of spring disasters in North East China. Second, the increase in the amount of spring rainfall further increases the severity of spring floods, resulting in increased SM on the surface, the formation of stagnant water in low-lying areas, and delays in spring plowing (especially in the Songnen Plain). Other studies have shown that Mongolia’s high-pressure systems and subtropical high-pressure belts, the main wind circulation of the westerly belt and the southwest water vapor transmission rate have all been weakened. We have greatly improved the spatiotemporal monitoring of SM, and the trends and disaster distributions based on the downscaled SM could provide references for the creation of climate change policies and monitoring of extreme weather.

## 5. Discussion and Conclusions

In this paper, an SFDM is constructed using passive microwave SM data and MODIS LST/NDVI data, and a time series reconstruction with the TSRDD method is used to ensure the continuity and consistency of long-term SM data sequences. By comparing the relative precision of different time series windows, the optimal window is selected to decompose the SM data with coarse spatial resolution into a high-resolution, continuous, monthly daytime and nighttime SM dataset (spanning 2002~2018, ~1 km). The in situ validation analysis resulted in R^2^ values of 0.61 and 0.73 for daytime and nighttime, respectively, and the average RMSE and bias values were 0.10 m^3^/m^3^ and 0.07 m^3^/m^3^ for daytime and 0.12 m^3^/m^3^ and 0.06 m^3^/m^3^ for nighttime, respectively. These results demonstrated that the method for downscaling the SM to 1-km resolution could reliably capture the variability in the daytime and nighttime soil water contents in North East China. We calculated the spatiotemporal distribution of the SM trends and statistically investigated the characteristics and patterns of droughts and floods in various regions of North East China with the SMA index. In the next step of this study, the response relationship between global meteorological phenomena and local disasters need to further quantitatively explored.

## Figures and Tables

**Table 1:** Definitions of disaster levels.

Disaster Type	SMA Values	Description
Slight waterlogging	90%–100%	The soil is very damp/extremely wet, and the color is dark
Moderate waterlogging	80%–90%	The soil is damp and brown, and there is water in low-lying places
Severe waterlogging	70%–80%	The soil is moist, and the color is brown
Normal	40%–70%	The soil moisture is within the normal range
Slight drought	30%–40%	Less soil moisture, and there is a risk for drought
Moderate drought	20%–30%	The soil is semidry
Severe drought	10%–20%	The soil is dry, and drought will occur

**Table 2:** The mean RMSE, MAE, and *R^2^* values between the corrected values of variable *z* and explanatory variables (original value) in the time series reconstruction of the difference decomposition (TSRDD) for different window sizes.

	Day			Night		
*w* ^1^	RMSE (m^3^/m^3^)	MAE (m^3^/m^3^)	*R^2^*	RMSE (m^3^/m^3^)	MAE (m^3^/m^3^)	*R^2^*
3	2.13	1.51	0.92	2.07	1.56	0.95
5	2.11	1.46	0.96	2.12	1.54	0.95
7	2.10	1.54	0.96	2.14	1.62	0.96
9	2.15	1.54	0.92	2.05	1.51	0.96
11	2.16	1.61	0.95	2.12	1.53	0.96
13	2.25	1.61	0.94	2.14	1.59	0.91
15	2.16	1.49	0.96	2.12	1.54	0.95
17	2.25	1.56	0.96	2.07	1.56	0.95
Average	2.16	1.54	0.95	2.10	1.56	0.95

^1^ *W* is the window size for the selected year from formula (8).
